# Vasculitis: From Target Molecules to Novel Therapeutic Approaches

**DOI:** 10.3390/biomedicines9070757

**Published:** 2021-06-30

**Authors:** Sang-Wan Chung

**Affiliations:** Division of Rheumatology, Department of Internal Medicine, School of Medicine, Kyung Hee University, 26 Kyungheedae-ro, Dongdaemun-gu, Seoul 02447, Korea; wanyworld83@gmail.com; Tel.: +82-2-958-8200

**Keywords:** systemic vasculitis, molecular target, novel treatment

## Abstract

Systemic vasculitis is a group of diverse diseases characterized by immune-mediated inflammation of blood vessels. Current treatments for vasculitis, such as glucocorticoids and alkylating agents, are associated with significant side effects. In addition, the management of both small and large vessel vasculitis is challenging due to a lack of robust markers of disease activity. Recent research has advanced our understanding of the pathogenesis of both small and large vessel vasculitis, and this has led to the development of novel biologic therapies capable of targeting key cytokine and cellular effectors of the inflammatory cascade. It is anticipated that these novel treatments will lead to more effective and less toxic treatment regimens for patients with systemic vasculitis.

## 1. Introduction

Systemic vasculitis pathologically denotes inflammation of a blood vessel, which is characterized by the presence of an inflammatory infiltrate and destruction of the vessel wall, causing stenosis and thrombosis. Vasculitis is a group of diverse disorders that demonstrate various organ involvement and clinical severity. Vasculitis can virtually affect any vessel in the organ system, and, depending on which vessel it invades, the manifestations can be very diverse. It is very common for highly variable conditions to lead to delays in diagnosis. Therefore, identifying vasculitis early, assessing response to therapy, and detecting disease relapse remain important clinical challenges [1,2].

As with many rheumatic diseases, there are no disease-specific clinical features or laboratory tests for making a definite diagnosis. Instead, vasculitis is classified according to classification criteria, of which the most widely used is the nomenclature, published by Chapel Hill Consensus Conference (CHCC) in 2012. Here, vasculitis is classified according to the size of the affected vessel: small vessel vasculitis (SVV), medium vessel vasculitis (MVV), and large vessel vasculitis (LVV) [3]. The epidemiology of systemic vasculitides varies greatly according to the type of vasculitis and the patient’s age, sex, and geographic location [4].

The pathogenesis of vasculitis remains unclear. One explanation is that exposure to an unidentified antigen, such as a virus, toxin, or cryptic epitope, leads to activation of the immune response. In some people, this immune response is not down-regulated, leading to the production of immune complexes that deposit in blood vessel walls and lead to vasculitis [5]. Pauci-immune vasculitides are not immune-complex-mediated and typically associate with antineutrophil cytoplasmic autoantibodies (ANCA), which are hypothesized to cause vascular damage indirectly by priming neutrophils to degranulate and to produce oxygen-free radicals.

The last decade has seen major advances in our understanding of the pathogenesis of vasculitis. These discoveries have led to the development of novel treatments, which seek to provide greater efficacy and a more acceptable side effect profile. In this review, we discuss the recent advances in understanding the pathogenesis of primary systemic vasculitides and the development of novel treatments.

## 2. Systemic Vasculitis Classification

Classification criteria are intended to create homogeneous patient groups for research. The classification systems for vasculitis are limited by overlapping features of subgroups and unrecognized pathogenic mechanisms. The most used classification criteria are defined by the size of the vessel they predominantly affect, namely, small, medium, or large, or variable vessel size (Table 1). The Chapel Hill International Consensus Conference (CHCC) of 2012 defined and standardized the nomenclature of systemic vasculitides [3].

LVV involves the aorta and its major branches, and includes giant cell arteritis (GCA) and Takayasu arteritis (TA). MVV involves the main visceral arteries and veins and their initial branches and includes polyarteritis nodosa (PAN) in adults. Kawasaki disease—the other major form of MVV and an acute arteritis of childhood—is not covered in this review. SVV involves arterioles, capillaries, intraparenchymal arteries, venules, and some veins and includes ANCA-associated vasculitis (AAV), the most common SVV in adults. There is, however, some overlap, and arteries of any size can potentially be involved in any case of the three main categories of dominant vessel pattern involvement [3]. In addition to the multi-organ systemic vasculitides, other forms of vasculitis have also been defined, such as single-organ vasculitis, including cutaneous arteritis, primary central nervous system vasculitis, and isolated aortitis; vasculitis associated with systemic disease, including rheumatoid vasculitis, lupus vasculitis, and sarcoid vasculitis; and vasculitis associated with an underlying cause: disease-related (Hepatitis B, Hepatitis C-associated cryoglobulinaemia, and cancer), or drug-related vasculitis [3].

The central feature of LVV is granulomatous arteritis. GCA exclusively affects individuals aged >50 years with a female-to-male predominance of 3:1. Additionally, GCA is more common in patients of Northern European descent than in Asian ethnic groups. GCA typically affects the branches of carotid, vertebral, and temporal arteries resulting in the classic symptoms of headache, jaw claudication, and loss of vision [6]. In contrast, TA usually affects females during the second and third decades of life. It is rare in Northern Europe but is more common in southeast Asia [7]. TA typically involves the aorta and its primary branches leading to vascular occlusion with claudication, aneurysm formation, aortic insufficiency, and cardiac failure. Current treatment of LVV is glucocorticoids [8]. Although methotrexate and azathioprine have been used as steroid-sparing agents, their effectiveness has not been proven in randomized controlled trials (RCT).

PAN is uncommon, with an estimated incidence of 1 to 10 per million. Both sexes are affected equally, and the peak age range of onset is between 40 and 60 years. The etiopathogenesis of PAN is strongly linked to viral hepatitis infection, particularly hepatitis B virus, which comprised over one-third of 348 PAN cases in the largest case series to date [9,10]. About 35% of polyarteritis nodosa (PAN) cases are associated with hepatitis B [11]. The incidence of hepatitis B virus-related PAN has declined substantially over the last four decades after improvements in immunization, transfusion practice, and hepatitis B virus therapy. PAN is characterized by a transmural necrotizing arteritis of muscular arteries [12]. The most commonly affected sites are the skin (causing livedo reticularis and ulceration) and peripheral nerves (leading to a mononeuritis multiplex). Involvement of visceral vessels is also common with multiple irregular arterial stenoses and microaneurysms demonstrable on contrast angiography in up to 90% of patients [13] with long-term immunosuppression with glucocorticoids alongside other agents such as cyclophosphamide, methotrexate, or azathioprine, improves patient outcomes, and supports an autoimmune component to pathogenesis [14].

Antineutrophil cytoplasmic antibody (ANCA)-associated vasculitides (AAV) are a group of systemic autoimmune disorders that predominately affects the small vessels. AAV are necrotizing vasculitides that are differentiated from other small vessel vasculitis by the lack of significant immune deposition in the vessel walls. AAV includes microscopic polyangiitis (MPA), granulomatosis with polyangiitis (GPA), and eosinophilic granulomatosis with polyangiitis (EGPA). The autoantibodies that define AAV are myeloperoxidase (MPO)-ANCA and proteinase 3 (PR3)-ANCA [15]. AAV are rare autoimmune conditions with a combined estimated prevalence of 46–184 per million [3]. However, they are associated with significant mortality. GPA mortality is reported to be greater than 90% at two years if left untreated [16,17]. Fortunately, the introduction of effective therapeutics has dramatically decreased the two-year mortality rate to 15% [18]. The treatment of AAV consists of remission induction followed by a maintenance phase. The treatment recommendations for induction or maintenance AAV vary based on the severity of the disease [16]. There are different definitions for determining what constitutes severe disease, but, generally, any AAV that is life- or organ-threatening is considered severe [19]. Current treatment options are effective; however, they are associated with significant patient morbidity due to treatment-related adverse effects.

Behçet disease (BD) is a rare relapsing, multisystem vasculitis, characterized by recurrent attacks of oral-genital ulcers and ocular, musculoskeletal, vascular, central nervous system (CNS), and gastrointestinal (GI) involvement. The prevalence of BD varies widely by geographic area, but, according to a recent meta-analysis, it is approximately 10.3 per 100,000 inhabitants [20]. The vascular involvement is the most frequent cause of mortality, and ocular involvement is the most important factor of morbidity in BD as it can cause blindness [21]. Treatment of BD is based on clinical manifestations. While colchicine, nonsteroidal anti-inflammatory agents, and topical treatments are often sufficient for mucocutaneous and joint involvement, immunosuppressive agents are required for major organ involvement [22].

As the understanding of the pathophysiology of systemic vasculitis increases, new therapies with fewer toxic effects are being proposed. This article will provide a review of current treatment options and an expert opinion on the future of AAV treatment.

## 3. Drug Discovery and Potential Targets in Vasculitis

**Figure 1:**
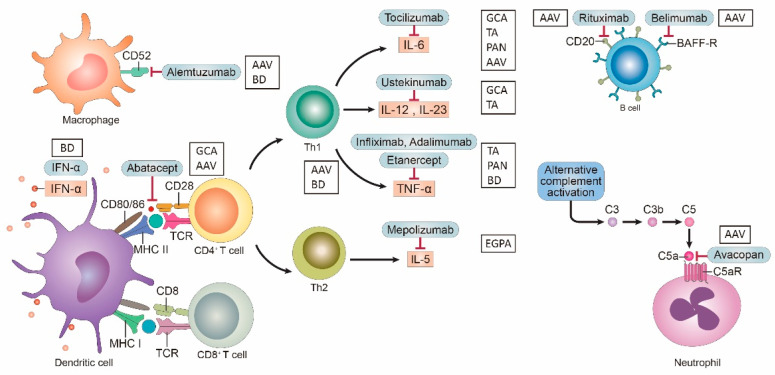
Targets and relative agent in vasculitis. IL, interleukin; mAb, monoclonal antibody; GCA, giant cell arteritis; TA, Takayasu‘s Arteritis; PAN, polyarteritis nodosa; TNF, tumor necoris factor; EGPA, Eosinophilic granulomatosis with polyangiitis, AAV, ANCA associated vasculitis; BAFF, B-cell activating factor; PDE, phosphodiesterase; IFN, interferon.

Treatment of the various type of vasculitis mainly relies on corticosteroids and conventional immunosuppressive drugs, such as methotrexate or azathioprines. Since vasculitis is a complex, chronic inflammatory disease, treatment may be needed for many different inflammatory molecules and targets. At present, research on these molecules and targets is mainly based on a few antibodies or inhibitors. Recent advances in the era of biologic agents have improved the management of difficult-to-treat cases dramatically. (Table 2, Figure 1).

### 3.1. Th1 Cytokines and Relative Drug Discovery

#### 3.1.1. IL-6

IL-6 plays a pathological effect on the inflammatory response in both the vessel wall and the systemic circulation. Tocilizumab is a humanized monoclonal antibody that competitively inhibits IL-6 by binding to circulating and membrane-bound IL-6 receptors. The first reported randomized controlled trial on the efficacy of tocilizumab in GCA randomized 20 patients to either 8 mg/kg tocilizumab delivered intravenously each month or placebo infusions in addition to glucocorticoids and found a higher relapse-free survival in the tocilizumab group (85 versus 20%, *p* = 0.001) at week 52 [23]. The effects of tocilizumab on glucocorticoid-sparing were observed in both relapsing and newly diagnosed GCA. The phase 3 Giant Cell Arteritis Actemra (GiACTA) trial enrolled 251 patients with new-onset GCA, randomized to one of four arms: tocilizumab 162 mg weekly or every other week (combined with a 26-week prednisone taper), or a prednisone taper alone (either 26 or 52 weeks). This study reported that Tocilizumab is an effective glucocorticoid-sparing therapy, demonstrating sustained glucocorticoid-free remission in 56% of patients receiving weekly tocilizumab compared with 18% of patients receiving a 52-week prednisone taper [24]. Tocilizumab is Food and Drug Administration (FDA)-approved for treatment of GCA.

In TA, a phase 3 trial about the effect of tocilizumab, Takayasu arteritis treated with tocilizumab (TAKT) was reported in 2017 [25]. Here, 36 relapsing TA patients were randomized to either tocilizumab, 162 mg weekly or placebo given weekly alongside a tapering glucocorticoid dose. Analyzed by an intention-to-treat method, tocilizumab failed to show difference in time to relapse as compared to placebo (hazard ratio [HR] 0.41, 95% confidence interval [CI] 0.15–1.10, *p* = 0.0596). However, the per-protocol analysis showed a significant difference for tocilizumab (*n* = 16) versus placebo (*n* = 17) (HR 0.34, 95% CI 0.11–1.00, *p* = 0.03). In 2020, the long-term efficacy and safety of tocilizumab in TA was reported. In that study, 28 patients received tocilizumab for 96 weeks. 46.4% of these 28 patients treated with tocilizumab reduced their dose to <0.1 mg/kg/day, thus showing evidence of a steroid-sparing effect of Tocilizumab in TA in long-term treatment [26].

There is no RCT for the effect of tocilizumab in PAN yet. In a recent case report, tocilizumab was effective for hepatitis B virus related PAN without Hepatitis B virus reactivation [27]. In a literature review based on 11 case reports, tocilizumab is effective in cases of refractory or relapsing polyarteritis nodosa and showed its glucocorticoid-sparing effect [28].

There are several case reports describing patients with AAV treated with tocilizumab showing that complete and sustained remission was achieved in many of the patients with refractory disease [29,30]. RCTs may be warranted in the future.

#### 3.1.2. IL-12 and IL-23

IL-23 is a pro-inflammatory cytokine composed of two subunits, IL-23A (p19) and IL-23B (p40), the latter shared with IL-12. The IL-23/IL-17 axis mainly plays a protective role against bacterial infections; its dysregulation plays a role in in immune-mediated inflammatory disorders [31,32,33]. As it has been reported that the IL-12/Th1 cell/IFN-γ pathway is involved in granulomatous inflammation in the pathogenesis of GCA, treatments targeting IL12 have been attempted, and the use of ustekinumab to treat LVV has been reported [34].

Ustekinumab is a monoclonal antibody that targets the p40 subunit of IL-12/23. One open-label study of 25 patients with refractory GCA treated with ustekinumab in addition to glucocorticoids demonstrated that no patients relapsed over 52 weeks. The median prednisolone dose decreased from 20 to 5 mg, and about 25% of patients were able to stop glucocorticoids. In addition, CT angiography showed an improvement in mural thickness with complete resolution in eight patients who underwent CT angiography before and after treatment [35]. However, in a recently reported prospective study, 10 out of 13 (77%) patients who failed to achieve the primary endpoint with ustekinumab in prednisone taper, and seven experienced disease flares after a mean period of 23 weeks [36]. Further research on the effect of Ustekinumab in GCA seems warranted.

Ustekinumab treatment in TA has been reported sporadically, and only in a few case series. One series of three patients with refractory TA treated with ustekinumab reported stabilization of clinical disease activity and normalization of inflammatory markers [37]. Recently, the results of a long-term follow-up on the same three patients reported that ustekinumab showed marginal effects on reducing prednisolone dose, and 2 of 3 patients discontinued ustekinumab treatment because of relapse and secondary failure [38].

#### 3.1.3. Tumor Necrosis Factor (TNF) α Inhibitor

TNF α inhibitors were the first biologic agents tried in various vasculitides. TNF α is an important cytokine for the formation of granuloma [39], and also for activation of endothelial cells [40].

After a few cases showing successful anti-TNF-α treatment in GCA patients had been reported, a comparative double-blind study was attempted using infliximab but was subsequently stopped due to the lack of efficacy on the prevention of relapse [41]. Using etanercept, a randomized controlled study showed a significant steroid sparing effect after one year in 17 patients, however not for a longer period [42]. Adding adalimumab to a standard prednisone regimen showed no steroid sparing effect in 70 patients in a 10-week prospective randomized controlled study [43]. As a result of these studies, anti-TNF-α therapy is not recommended in GCA.

Several retrospective studies and case series reported that TNF α inhibitors were effective in most patients with refractory Takayasu’s arteritis [44,45,46]. A two-year follow-up cohort study from Norway reported higher rates of sustained remission as well as lesser progression of angiographic lesions in patients receiving anti-TNF-α agents, when compared with conventional treatments in Takayasu’s arteritis [47].

In PAN, infliximab has been used in refractory forms of the disease or because of intolerance to conventional drugs and seems to be effective [48]. In a small case series, nine refractory PAN patients were treated with infliximab and 8 of 9 patients (89%) achieved significant improvement and prednisone dose reduction of 50% [49].

A number of open-label studies and case series have reported the usefulness of anti-TNF-α therapies in AAV, although these results have not been confirmed in RCTs. In the Wegener’s Granulomatosis Etanercept Trial (WGET), which recruited 174 patients with GPA, there was no benefit from etanercept on the sustained remission rate [50]. With little evidence for its effectiveness, the use of anti-TNF α treatment in AAV may be significantly limited in the future. Similarly, for EGPA, the only available information is derived from five case reports with conflicting findings that do not support anti-TNF-α use to treat EGPA [51,52,53].

In small RCT of 40 patients with BD, etanercept was significantly more effective in suppressing most of the mucocutaneous manifestations, such as oral ulcers and erythema nodosum, than placebo [54]. Several observational studies and case series also confirmed the beneficial effects of infliximab and adalimumab on mucocutaneous lesions of BD [55]. Most of the studies on the effects of TNF-α inhibitors in BD are in ocular manifestations and mainly reported in case series. Infliximab significantly decreases in relapse rate and glucocorticoid dosage in BD patients with ocular involvement [56,57,58]. In the first prospective study in 63 patients with BD uveitis, uveoretinitis improved with infliximab treatment in 92% and maintained for up to 12 months [59]. A 1-year observational multicenter study reported the results of infliximab and adalimumab use in 124 patients with refractory BD uveitis, and complete remission was achieved in 84/124 (68%) [60]. A recent retrospective observational study also reported that adalimumab was highly effective and safe for treatment of BD related uveitis [61]. An open-label study of 177 patients with BD related uveitis compared the efficacy of infliximab (103 patients) versus adalimumab (74 patients) as a first-line biologic agent. In this study, an improvement in all ocular parameters were observed in both groups after 1-year treatment; however, adalimumab had significantly better ocular outcomes in some parameters [62]. In BD related vascular manifestation, such as deep vein thrombosis, superficial thrombophlebitis, a retrospective study reported that adalimumab achieved significantly higher vascular response (34/35, 97%) compared with conventional immunosupressants (23/35, 66%) during a mean follow-up of 25.7 ± 23.2 months. Significantly lower vascular relapse was also observed in adalimumab group [63]. In recent two multicenter observational studies, clinical remission was achieved in 89% and 80% of patients with BD, respectively, with vascular involvement refractory to conventional ISs treatment [64,65].

### 3.2. Th2 Cytokines and Relative Drug Discovery

#### IL-5

IL-5 is the major cytokine responsible for eosinophil activation, chemoattraction, and survival. Several studies have reported elevated serum IL-5 levels in EGPA [66,67]. Recently, IL-5 antagonists have been studied as an EGPA-specific treatment. Mepolizumab, a humanized anti-IL-5 monoclonal antibody, selectively inhibits eosinophilic inflammation and is approved to treat severe eosinophilic asthma [68]. A benefit of mepolizumab treatment in EGPA patients was observed in previous small open-label pilot studies [69,70,71]. In the large scale randomized controlled trial, 136 EGPA patients with an uncontrolled disease injected 300 mg of mepolizumab once a month subcutaneously. The remission rate in patients treated with mepolizumab was significantly higher than that of patients treated with placebo (28% vs. 3%, of the participants had ≥24 weeks of accrued remission; odds ratio, 5.91; 95% confidence interval [CI], 2.68 to 13.03; *p* < 0.001) [72]. This study led to FDA authorization of mepolizumab as the first drug specifically approved for EGPA.

### 3.3. Targets and Drug Discovery of B Cells

#### 3.3.1. CD20

B cells are clearly central to the pathogenesis of AAV, as they produce ANCAs. Rituximab is a chimeric monoclonal antibody that induces B-cell depletion by binding CD20 expressing B cells. Its development has led to advances in AAV therapy. The Rituximab in ANCA-Associated Vasculitis (RAVE) [73] and Rituximab Versus Cyclophosphamide in ANCA-Associated Vasculitis (RITUXVAS) [74] trials established noninferiority of rituximab to cyclophosphamide for AAV remission induction. Several second-generation anti-CD20 drugs have been developed, one of which, ofatumumab, has been tested in one small case series of patients with AAV, with results showing its therapeutic benefit. However, there has been no RCT yet [75].

#### 3.3.2. BAFF

B-cell activating factor (BAFF), also known as B-lymphocyte stimulator (BlyS), plays an important role in B cell maturation and is increasingly recognized as important in the pathogenesis of relapsing AAV. Increased BAFF expression is evident in patients with active vasculitis, and preclinical data suggest that high BAFF concentrations can promote the survival of autoreactive B cells that, under normal conditions, would be degraded [76,77]. Belimumab is a fully humanized monoclonal antibody that binds to BAFF receptors on B cells. It is licensed for the treatment of systemic lupus erythematosus [78,79].

In AAV, the Belimumab in Remission of Vasculitis (BREVAS) trial examined the addition of belimumab to azathioprine and glucocorticoids for maintenance of remission in patients with GPA and MPA [80]. However, the trial was stopped early due to suboptimal recruitment, and no improvement in the relapse rate was observed. The combination of rituximab and belimumab is being investigated further in an ongoing randomized double-blind placebo-controlled trial, the Rituximab and Belimumab Combination Therapy in PR3-AAV trial (COMBIVAS) (ClinicalTrials.gov identifier: NCT03967925).

### 3.4. B-Cell and T-Cell Co-Stimulation and Depletion

#### 3.4.1. CD28–CD80/CD86

Co-stimulatory molecules predominantly modulate the immune responses by activating T- and B-cell functions, but also affect dendritic cell and macrophage functions and play a crucial role in inflammation. Abatacept, a fusion protein of the extracellular domain of CTLA-4 and the Fc fragment of human IgG1 (CTLA-4–Ig), is an inhibitor of T lymphocyte activation by means of co-stimulatory blockade by binding to CD80 and CD86 receptors on APC that is needed for antigen-presenting activation of T cells [81].

In GCA, 41 patients were randomized and relapse-free survival was 48% with abatacept as maintenance therapy compared with 31% with placebo (*p* = 0.049) [82]. The duration of remission is significantly longer in the abatacept group than that of the placebo group. In a double-blind randomized controlled multicenter study, 34 patients with TA were treated with abatacept at a dose of 10 mg/kg on days 1, 15, 29, and at 8 weeks. Patients attaining remission at 12 weeks were randomized to either receive placebo (*n* = 15) or monthly abatacept (*n* = 11) and followed up until 12 months. However, there was no difference in the duration of remission and relapse-free survival at 12 months between the two groups [83].

In AAV, infiltrations of granulomatous T-cells were observed in lungs and kidneys, suggesting a pathogenic role of T cells. In an open-label trial of 20 patients with a non-severe relapsing GPA who were treated with abatacept, the remission rate was about 80% and steroid discontinuation rate 75% [84]. The ongoing phase III randomized placebo-controlled Abatacept for the Treatment of Relapsing, Non-Severe, Granulomatosis with Polyangiitis (ABROGATE) (ClinicalTrials.gov identifier: NCT02108860) trial is currently recruiting patients.

#### 3.4.2. CD52

CD52 is expressed on monocytes, macrophages, and eosinophils. Depletion of B cells and T cells can be achieved by alemtuzumab [85]. This humanized anti-CD52 monoclonal antibody selectively depletes lymphocytes and has been shown to be effective in other systemic vasculitides such as Behçet’s disease [86]. Alemtuzumab as remission induction therapy was effective in 84% of 32 BD patients, and sustained remission was achieved in 69% at 12 months [86]. Walsh et al. reported results of a retrospective long-term study of 71 patients with refractory or relapsing AAV treated with alemtuzumab and found it useful to achieve remission with a lower relapse rate [87]. A randomized, prospective, open-label study of alemtuzumab for remission induction in refractory AAV (ALEVIATE trial) was presented in the form of an abstract in 2019. In this study, remission was achieved in 65% of AAV patients at 6 months and 35% sustained remission at one year [88] However, adverse events, such as infection, are high compared to standard treatment.

### 3.5. Targeting Complement

#### C5a Receptors

The complement system is a central mediator of antibody-mediated immune responses. C5 is a potent effector protein in this pathway, exerting its effects through its cleavage products: C5a, a potent anaphylatoxin and chemoattractant, and C5b, part of the a membrane attack complex that lyses target cells [89]. In patients with AAV, complement deposition is evident at sites of tissue inflammation, such as kidneys, and high plasma levels of complements correlate with disease severity [90,91].

Avacopan contains a small molecule that binds to C5a preventing it from binding to its receptor. Clinical trial results of C5a receptor inhibition with avacopan have shown promising results [92]. Sixty-seven patients with AAV were randomized to either high-dose glucocorticoids, avacopan plus low-dose glucocorticoids, or avacopan alone alongside cyclophosphamide or rituximab induction. At 12 weeks, a 50% reduction from baseline in the Birmingham Vasculitis Activity Score (BVAS) occurred in 86% of the avacopan/glucocorticoid and 81% of the avacopan-alone groups, compared with 70% in the glucocorticoid group (*p* = 0.002 and *p* = 0.01, respectively). However, this study included only non-severe disease. The results of a phase III trial, the Avacopan in Patients With ANCA-Associated Vasculitis (ADVOCATE) trial, were reported in 2021 [93]. This study enrolled 331 patients, randomized to receive either avacopan or glucocorticoids during remission induction with either cyclophosphamide or rituximab. At 26 weeks, the number of patients in remission, as assessed by a score 0 on the BVAS and withdrawal of steroid therapy, was not inferior in both the avacopan and prednisone groups (72.3%, 70.1% respectively). Additional data showed that avacopan was superior over glucocorticoids in sustained remission at 52 weeks, with an acceptable safety profile.

An anti-C5a monoclonal Ab, IFX-1 is also being evaluated in phase II studies (INFLARX trial, NCT03895801 and NCT03712345). Recruitment is ongoing and completion is estimated by July 2021.

### 3.6. Other Targets

#### Interferon-α

Interferons (IFN), a large family of glycoproteins, produce a cellular response to the microbes, tumors, and antigens [94]. IFN-α was demonstrated to modulate the Th1/Th2 balance toward Th1 by increased IFN-γ production and inhibiting IL-5 and IL-13 production in Th2 cells [95,96].

The efficacy of IFN-α has been well established in BD, with the data coming from case series, especially in ocular manifestations [97,98,99]. A retrospective study reported that there was no difference between azathioprine plus colchicine and IFN-α2a treatment in BD uveitis regarding remission and relapse rates [100]. Some case reports also reported the efficacy of INF-α in neuro BD [101,102]. In an RCT of 44 patients of BD, IFN-α treatment significantly improved mucocutaneous manifestations, such as orogenital ulcers, and papulopustular lesions [103]. In a recent prospective study of 33 patients with deep venous thrombosis, one of the serious complications of BD, the relapse rate was lower and recanalization rate was higher in patients treated with IFN-α compared with AZA (12% vs. 45% and 86% vs. 45%) [104].

**Table 2:** Targets and relative agent in vasculitis.

Target		Agent	Vasculitis	References
Th1 cytokines	IL-6	Tocilizumab (anti-IL-6R mAb)	GCA, TA PAN AAV	[23,24] [25,26] [27,28] [29,30]
	IL-12 and IL-23	Ustekinumab (p40 subunit of IL-12/IL-23 mAb)	GCA TA	[35] [37,38]
	TNF-α	Infliximab, Adalimumab (anti-TNF-α mAb) Etanercept (TNF-α receptor fusion protein)	TA PAN BD	[44,45,46,47] [48,49] [54,55,56,57,58,59] [60,61,62,63,64,65]
Th2 cytokines	IL-5	Mepolizumab (anti-IL-5 mAb)	EGPA	[69,70,71,72]
B cells	CD20	Rituximab (anti-CD20 mAb)	AAV	[73,74]
	BAFF-R	Belimumab (BAFF-receptor mAb)	AAV	[80]
Co-stimulatory molecules	CD28–CD80/CD86	Abatacept (CTLA4Ig fusion protein)	GCA AAV	[82] [84]
	CD52	Alemtuzumab (anti-CD52 mAb)	AAV BD	[87,88] [86]
Complement	C5a	Avacopan (C5a receptor inhibitor)	AAV	[92,93]
Other targets	IFN-α	IFN-α	BD	[97,98,99,100] [101,102,103,104]

## 4. Conclusions

As the understanding of the pathogenesis of systemic vasculitis advances, novel target molecules and therapeutic approaches are being proposed. Treatment outcomes of vasculitis have improved with several new evidence-based treatments.

In spite of the success of blocking IL-6 in large vessel vasculitis, relapse rates remain high, suggesting that further study is needed. In AAV, recent trials of therapies that target B-cell activation, complements, and IL-5 provide encouraging evidence of better outcomes for these patients. Future clinical trials of these novel therapeutic agents will need to establish their efficacy and, as an increasing number of potential treatments become available, will need to indicate how they can be used to complement or replace existing approaches.

## Figures and Tables

**Table 1:** Nomenclature of the systemic vasculitides defined during the 2012 International Chapel Hill Consensus Conference (Adapted from [3]).

**Systemic Vasculitis**
**Large-vessel vasculitis (LVV)**
Giant cell arteritis (GCA)
Takayasu arteritis (TA)
**Medium-vessel vasculitis (MVV)**
Polyarteritis nodosa (PAN)
Kawasaki disease (KD)
**Small-vessel vasculitis (SVV)**
**Anti-neutrophil cytoplasmic antibody (ANCA) associated vasculitis (AAV)**
Microscopic polyangiitis (MPA)
Granulomatosis with polyangitis (GPA)
Eosinophilic granulomatosis with polyangitis (EGPA)
**Immune complex vasculitis**
Anti-glomerular basement membrane (anti-GBM) disease
Cryoglobulinemic vasculitis (CV)
IgA vasculitis (Henoch-Schonlein) (IgAV)
Hypocomplementemic urticarial vasculitis
**Variable vessel vasculitis (VVV)**
Behçet’s disease (BD)
Cogan’s syndrome (CS)
